# Mental health and hypertension: assessing the prevalence of anxiety and depression and their associated factors in a tertiary care population

**DOI:** 10.3389/fpubh.2025.1545386

**Published:** 2025-05-09

**Authors:** Geetha Kandasamy, Thangamani Subramani, Mona Almanasef, Khalid Orayj, Eman Shorog, Asma M. Alshahrani, Alhanouf Alsaab, Zainah M. Alshahrani, Siyad Palayakkodan

**Affiliations:** ^1^Department of Clinical Pharmacy, College of Pharmacy, King Khalid University, Abha, Saudi Arabia; ^2^Department of Pharmacy Practice, Grace College of Pharmacy, Palakkad, India; ^3^Department of Clinical Pharmacy, College of Pharmacy, Shaqra University, Dawadimi, Saudi Arabia; ^4^Abha International Private Hospital, Abha, Saudi Arabia; ^5^General Directorate of Infection Prevention and Control, Ministry of Health, Riyadh, Saudi Arabia

**Keywords:** anxiety, depression, hypertension, HAD-A, HAD-D

## Abstract

**Background:**

Anxiety and depression are more common in those with hypertension. Identifying factors may lead to earlier assessment and treatment of depression and anxiety. So the study was aimed to assess the prevalence of anxiety and depression and examine the associated factors among hypertensive patients in a tertiary care hospital.

**Methods:**

This cross sectional study was conducted over a six-months period (October 2023 to March 2024) in the Department of Medicine at Karuna Medical College Hospital, Kerala, India. The Hospital Anxiety and Depression Scale (HADS) was used to assess the symptoms of anxiety and depression. Multivariate logistic regression analysis was used to identify factors associated with anxiety and depression.

**Results:**

Among 262 hypertensive patients, the prevalence of symptoms of anxiety were 43.8% and symptoms of depression were present in 51.3%. Female gender [OR 1.607 95% CI (0.962–2.687)] and BMI ≥ 25 [OR 0.608 95% CI (0.361–1.024)] were found to be significantly associated with anxiety (*p* < 0.05). Uncontrolled BP and stage1/stage 2 hypertension were found to be significantly associated with anxiety and depression (*p* < 0.05). Whereas factors like age (>50 years), living with alone, unmarried/widow, unemployed/retired/house wife, illiterate, living in rural area and patients with comorbidities showed to be non-significantly association with anxiety and depression (*p* > 0.05) in patients with hypertension.

**Conclusion:**

According to the findings, overall 50% of hypertensive patients experience anxiety and depression, which were significantly associated with uncontrolled BP, stage1/stage 2 hypertension, female gender and obesity. Healthcare providers can help identify mental health issues early, improving outcomes, with special attention needed for women with comorbidities and limited social support.

## 1 Introduction

Globally, hypertension is a leading cause of death and a serious public health concern (1). Over 1.3 billion people worldwide suffer from hypertension, with 220 million adults in India alone suspected to have the condition. The number of patients with hypertension aged 30–79 years has increased twofold from 1990 to 2019 (2, 3). The control rate of hypertension in India is alarmingly low, and uncontrolled blood pressure (BP) is a major risk factor for both microvascular and macrovascular complications, as well as stroke, coronary heart disease, chronic kidney disease, retinopathy, and more (4, 5).

In addition to hypertension, mental disorders are a significant public health concern. A meta-analysis found that over 38% of individuals have both hypertension and anxiety (6). Anxiety, depression, and other psychological disorders are more common in those with hypertension (7–10). Individuals with hypertension experience a wide range of intense emotions, which increase their susceptibility to mental health problems, particularly depression and anxiety (11). According to studies conducted in India, the prevalence of depression was found to be 49%, with approximately 53.4% of hypertensive individuals with normal blood pressure also suffering from depression. Furthermore, significant depression was found in approximately 44.6% of patients with pre-hypertension and 44.6% of patients with stage II hypertension (12, 13).

The connection between anxiety and hypertension is complex, and studies have shown that dopamine and associated neurotransmitters exhibit antihypertensive effects. A deficiency of dopamine in specific brain regions may result in elevated BP and depression (14). Anxiety typically increases blood lipids, sympathetic activity, plasma renin activity, systemic vascular resistance, and disrupts homeostasis (15). There are multiple pathways through which anxiety, driven by ongoing stress, anger, and depression, can contribute to cardiovascular disease (16).

Many individuals diagnosed with hypertension face significant challenges, such as symptoms of illness, reduced quality of life, and role impairment. These factors may increase their susceptibility to psychological disturbances, particularly depression (17). Depression among hypertensive individuals is associated with higher medical care expenses compared to hypertension alone. Furthermore, the risk of all-cause mortality is 15% higher for patients with both depression and hypertension than for those with depression alone (18). The overwhelming symptoms of anxiety and depression can impair a person’s quality of life and interfere with daily functioning. Several factors, including residence, lack of social support, body mass index, income, and low levels of education, influence the prevalence of anxiety and depression in patients with chronic illnesses (19). Patients with hypertension have a higher incidence of depression than the general population. Depression has been associated with factors such as female gender, low socioeconomic status, not living with a spouse, lack of physical activity, low educational level, and uncontrolled BP (20).

The correlation between depression and hypertension is not yet fully established. Some studies suggest that individuals with hypertension may be more prone to depression (21). Patients with both depression and hypertension are less likely to adhere to their treatment plans, make lifestyle changes, or attend follow-up appointments (22). The prevalence of depression and anxiety varies according to the stages of hypertension and tends to increase as hypertension progresses. Another concern is the high rate of undiagnosed depression in individuals with hypertension, indicating that patients should undergo psychiatric evaluations to identify symptoms of depression (23).

Identifying factors that contribute to depression and anxiety may lead to earlier assessment and treatment. However, in developing countries like India, the factors associated with anxiety and depression in patients with hypertension have not been sufficiently evaluated. The primary hypothesis of the study is that hypertensive patients in a tertiary care hospital are more likely to experience higher levels of anxiety and depression compared to the general population, with sociodemographic, clinical, and lifestyle factors serving as significant predictors of these mental health conditions. Therefore, this study aimed to assess the prevalence of anxiety and depression and examine the associated factors among hypertensive patients in a tertiary care hospital. The findings could inform targeted interventions, improve healthcare practices, and enhance mental health support in tertiary care settings, ultimately promoting the well-being of hypertensive patients and more holistic care.

## 2 Methodology

### 2.1 Study design and sample size

**Figure 1:**
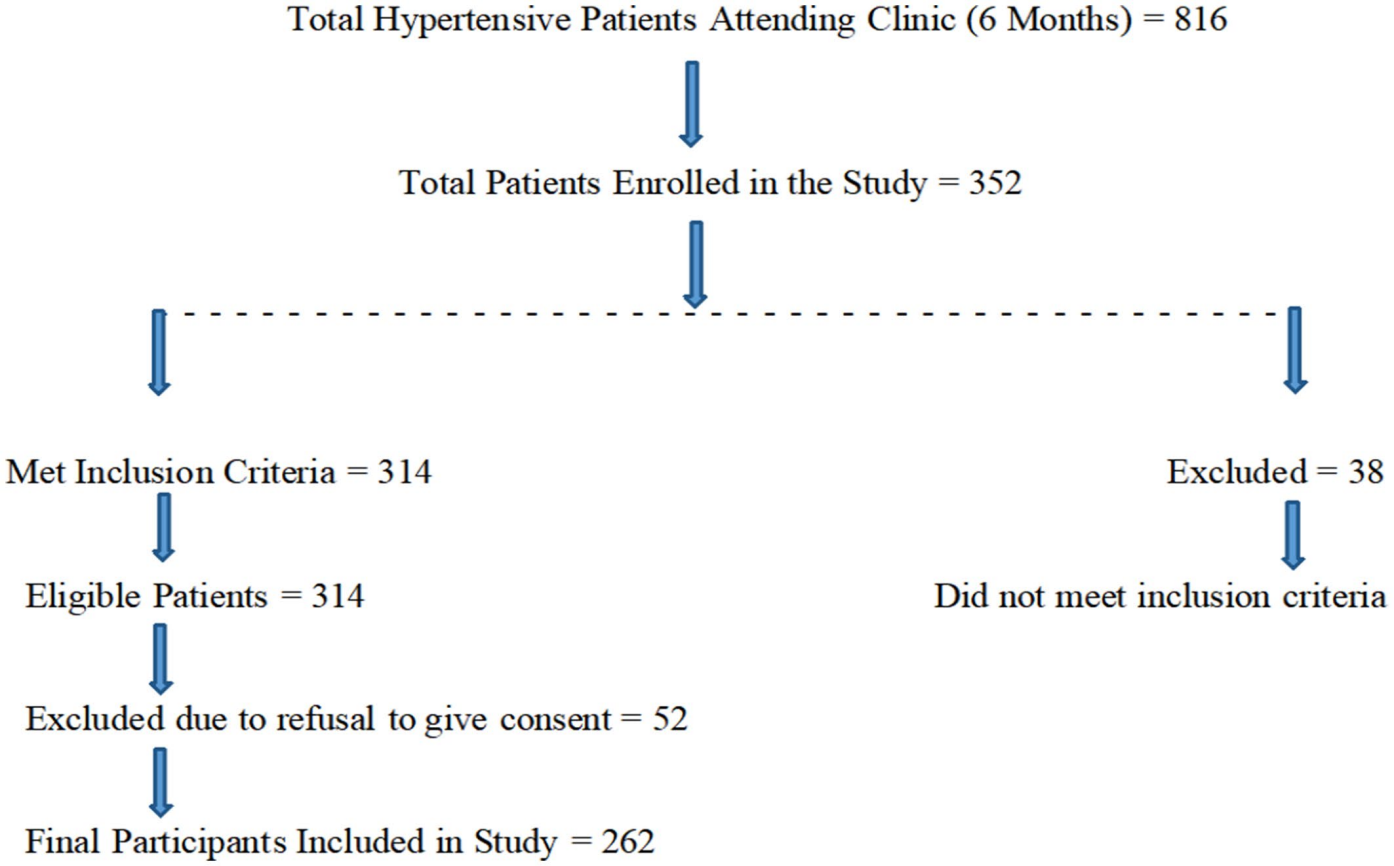
Study design.

This cross-sectional study was conducted over a six-month period, from October 2023 to March 2024, in the Department of Medicine at Karuna Medical College Hospital, Kerala, India. Approximately 34 hypertensive patients were seen each week, resulting in a total of around 816 patients over 6 months. Using a sample size calculator, a representative target sample size was determined to meet the research objectives and ensure adequate statistical power. Based on a population size of 816 participants, a response rate of 50%, a confidence level of 95%, and a margin of error of ±5%, the estimated sample size was 262, calculated using the Raosoft calculator (24) (Figure 1).

### 2.2 Population criteria

Hypertensive patients who were on regular treatment and provided informed consent were included in the study. Patients with hypertension who had been diagnosed with other chronic conditions such as stroke, coronary artery disease, asthma, chronic kidney disease, or newly diagnosed hypertension were excluded. Additionally, hypertensive patients with a history of pregnancy, renal diseases, cancer, dementia, depression, or other psychiatric disorders, as well as those currently undergoing treatment with antidepressants, antipsychotics, or anxiolytics, were also excluded.

### 2.3 Data collection

Prior approval was obtained from the Institutional Review Committee (IRC) of Karuna Medical College Hospital. Written informed consent was obtained from all participants. Data were collected using a predesigned data entry form. Demographic details, including sex, age, residence, occupation, monthly income, educational level, marital status, smoking habits, and alcohol intake, were collected through face-to-face interviews. Clinical characteristics, such as comorbid conditions, the number of antihypertensive medications, and the duration of hypertension, were obtained from patients’ case sheets. Blood pressure (BP) was measured using a sphygmomanometer and stethoscope by trained medical staff. BP measurements were taken three times on the patient’s left arm after 15 min of seated rest. The average of the last two of the three BP readings was used to calculate each participant’s BP.

### 2.4 Hospital Anxiety and Depression Scale (HADS)

The Hospital Anxiety and Depression Scale (HADS), developed by Zigmond and Snaith in 1983, was used to assess psychiatric morbidity, specifically anxiety and depression. HADS is a 14-item self-report tool designed to identify anxiety and depression in outpatient general medical populations. It consists of 14 questions: seven assess symptoms of anxiety (HAD-A), and seven assess symptoms of depression (HAD-D). The Malayalam version of the HADS was validated and found to have good internal consistency, with a Cronbach’s alpha value of 0.834. This indicates that the scale is reliable and suitable for assessing anxiety and depression among the target population in this region. Each question is scored on a scale from 0 to 3, and the sums of the anxiety and depression subscales are calculated independently. Scores for each subscale range from 0 to 21, with items scored using a 4-point Likert scale. The total score ranges from 0 to 42, with categories defined as follows: normal (0–7), mild (8–12), moderate (11–14), and severe (15–21). Participants with scores of 8 or higher on either subscale were considered at risk for anxiety and/or depression (25, 26).

The JNC 7 (Seventh Report of the Joint National Committee on Prevention, Detection, Evaluation, and Treatment of High Blood Pressure) classifies hypertension as follows: normal systolic BP (SBP) < 120 mm Hg and diastolic BP (DBP) < 80 mm Hg; prehypertension with SBP 120–139 mm Hg and DBP 80–89 mm Hg; stage I hypertension with SBP 140–159 mm Hg and DBP 90–99 mm Hg; and stage II hypertension with SBP ≥ 160 mm Hg and DBP ≥ 100 mm Hg (27).

### 2.5 Ethical approval

Ethical clearance (KMC/IHEC/11/2024) was obtained from the Institutional Ethics Committee of Karuna Medical College, Kerala, India, prior to the commencement of the study. All participants were provided with detailed information about the study and gave written informed consent. Data protection and confidentiality were maintained by anonymizing participant data and securely storing all information in restricted access systems.

### 2.6 Statistical analysis

The data were analyzed using SPSS (Statistical Package for the Social Sciences) version 20. Categorical variables between patients with and without symptoms of anxiety or depression were compared using the chi-square test. Multivariate logistic regression analysis was conducted to identify factors associated with anxiety and depression. A *p*-value of <0.05 was considered statistically significant, and the odds ratio (OR) with a 95% confidence interval (CI) was calculated.

## 3 Results

### 3.1 Sociodemographic characteristics of study population

A total of 262 hypertensive patients were included in the study based on the inclusion criteria. Among them, 132 (50.4%) were female, and 130 (49.6%) were male. Patients aged over 60 years 141 (53.8%) were more common than those under 60 years 121 (46.2%). Additionally, 182 (69.5%) of the patients resided in rural areas (Table 1).

**Table 1:** Baseline characteristics of study population.

S. No	Parameters		No. of patients (*N* = 262)	Percentage of patients (%)
1.	Gender	Male	130	49.6
		Female	132	50.4
2.	Age	31–40	09	3.5
		41–50	36	13.7
		51–60	76	29
		>60	141	53.8
3.	Marital status	Married	248	94.6
		Unmarried	8	3
		Divorced/widowed	6	2.3
4.	Occupation	Employed	68	26
		Unemployed	43	16.4
		House wife	87	33.2
		Retired	64	24.4
5	Place of residence	Rural	182	69.5
		Urban	80	30.5
6	Education	Primary school	127	48.4
		Secondary school	77	29.3
		College and above	37	14.1
		No formal education	21	8
7	Family history of Psychiatry disease	Yes	34	13
		No	228	87
8	Hypertension status	Uncontrolled	153	58.4
		Controlled	109	41.6
9	Stages of hypertension	Normal/Prehypertension	62	23.66
		Stage1/Stage 2	200	76.33
10	BMI (kg/m^2^)	<18.5	7	2.6
		18.5–24.9	154	58.8
		25–29.9	88	33.5
		>30	13	4.9
11	Alcohol use	Yes	52	20
		No	210	80
12	Smoking	Yes	79	30.2
		No	183	69.8

### 3.2 Clinical, psychosocial parameters of study population

More than half of the patients with hypertension, 161 (61.4%), had a body mass index (BMI) of 18.5–24.9 kg/m^2^. A majority of the patients, 248 (94.6%), were married, while 4 (5.4%) were unmarried or divorced. Of the total, 68 (26%) were employed, while the remaining 201 (74%) were not employed, including housewives and retirees. Additionally, 34 (13%) had a family history of psychiatric disease, 79 (30.2%) were smokers, and 52 (20%) were alcohol consumers.

Table 2 shows the distribution of sociodemographic and clinical characteristics of hypertensive patients based on symptoms of anxiety and depression assessed using HADS. A significantly higher number of hypertensive female patients, 79.8% (*p* < 0.05), had anxiety and depression compared to male patients. Among married patients, 87.5% had depression, and 76.8% had anxiety. Anxiety and depression were more common among patients aged >50 years, with 103 (88%) experiencing anxiety and 114 (83.2%) experiencing depression, compared to those aged ≤50 years, where 14 (12%) and 23 (16.8%) experienced anxiety and depression, respectively. There was a significantly higher percentage of patients with anxiety in the >50 years age group, 103 (88%), compared to the ≤50 years group, 14 (12%) (*p* < 0.05). Patients with no occupation, such as housewives, retirees, or the unemployed, were more commonly affected by anxiety, 88 (75.2%), and depression, 104 (75.9%). Those living in rural areas were significantly more affected by symptoms of depression, 103 (75.2%) (*p* < 0.05). Patients with uncontrolled BP levels were significantly more likely to have anxiety, 77 (65.8%), and depression, 94 (68.6%) (*p* < 0.05). A statistically significant association was observed between stages of hypertension and anxiety (*p* = 0.001), with 101 patients (86.3%) in Stage 1 or Stage 2 hypertension reporting higher anxiety symptoms compared to 16 patients (13.7%) in the normal or prehypertensive range. However, the association with depression was not statistically significant (*p* = 0.198), as 109 patients (79.6%) in Stage 1 or Stage 2 hypertension and 28 patients (20.4%) in the normal or prehypertensive range exhibited depressive symptoms. Patients with a BMI ≥ 25 were statistically more likely to have anxiety, 79 (67.5%) (*p* = 0.041).

**Table 2:** Distribution of factors based on symptoms of anxiety and depression in hypertensive patients by using HADS.

S. No	Parameters	Anxiety			Depression		
		Score< 10 (*n* = 145)	Score>10 (*n* = 117)	*p* value	Score< 10 (*n* = 125)	Score>10 (*n* = 137)	*p* value
1	Age			0.0446*			0.861
	≤ 50 years	31 (21.4)	14 (12)		22 (17.6)	23 (16.8)	
	> 50 years	114 (78.6)	103 (88)		103 (82.4)	114 (83.2)	
2	Sex			0.0244*			0.0484*
	Female	64 (44.1)	68 (58.1)		55 (44)	77 (56.2)	
	Male	81 (55.9)	49 (41.9)		70 (66)	60 (43.8)	
3	Marital Status			0.8892			0.708
	Married	137 (94.5)	111 (94.9)		119 (95.2)	129 (94.2)	
	Unmarried/Widow	08 (5.5)	06 (5.1)		06 (4.8)	08 (5.8)	
4	Employment status			0.6984			0.470
	Employed	39 (26.9)	29 (24.8)		35 (28)	33 (24.1)	
	Housewife/Retired/Unemployed	106 (73.1)	88 (75.2)		90 (72)	104 (75.9)	
5	Area of living			0.9408			0.0354*
	Rural	101 (69.7)	81 (69.2)		79 (63.2)	103 (75.2)	
	Urban	44 (30.3)	36 (30.8)		46 (36.8)	34 (24.8)	
6	Blood pressure			0.0282*			0.001*
	Controlled	69 (47.6)	40 (34.2)		66 (52.8)	43 (31.4)	
	Uncontrolled	76 (52.4)	77 (65.8)		59 (47.2)	94 (68.6)	
7	Stages of hypertension						0.198
	Normal/Prehypertension	46 (31.7)	16 (13.7)	0.001*	34 (27.2)	28 (20.4)	
	Stage1/Stage 2	99 (68.3)	101 (86.3)		91 (72.8)	109 (79.6)	
7	Body Mass Index (BMI) (kg/m^2^)			0.0419*			0.221
	<24.9	82 (56.6)	79 (67.5)		72 (57.6)	89 (65)	
	≥25	63 (43.4)	38 (32.5)		53 (42.4)	48 (35)	

*indicates *P* < 0.05 considered as significant.

Multivariate logistic regression analysis was performed to assess the factors associated with depression and anxiety among hypertensive patients (Tables 3, 4). The factors associated with anxiety are presented in Table 3. Female gender [OR 1.607, 95% CI (0.962–2.687)], BMI ≥ 25 [OR 0.608, 95% CI (0.361–1.024)] and stages of hypertension (stage1/stage2) [OR 14.328 95% CI (7.6606–26.801)] were found to be significantly associated with anxiety (*p* < 0.05) in hypertensive patients. However, other factors such as age >50 years [OR 1.792, 95% CI (0.847–3.790)], living alone [OR 0.581, 95% CI (0.138–2.441)], being unmarried or widowed [OR 1.932, 95% CI (0.631–5.917)], unemployed/retired/housewife status [OR 0.988, 95% CI (0.544–1.796)], illiteracy [OR 0.802, 95% CI (0.304–2.117)], living in a rural area [OR 0.826, 95% CI (0.462–1.477)], uncontrolled BP [OR 1.196, 95% CI (0.708–2.021)], and having comorbidities [OR 1.124, 95% CI (0.671–1.884)] were not significantly associated with anxiety (*p* > 0.05).

**Table 3:** Factors associated with anxiety symptoms in hypertensive patients.

S. No	Parameters	Anxiety (*n* = 117)	Confidence level	Odds ratio	*p* value
1	Age				
	≤50	14	Reference	Reference	
	>50	103	0.847–3.790	1.792	0.127
2	Gender				
	Male	49	Reference	Reference	
	Female	68	0.962–2.687	1.607	0.033*
3	Body mass Index (BMI) (kg/m^2^)				
	<24.9	79	Reference	Reference	
	≥25	38	0.361–1.024	0.608	0.041*
4	Marital Status				
	Married	111	Reference	Reference	
	Unmarried	6	0.138–2.441	0.581	0.459
5	Living Arrangement				
	with family	105	Reference	Reference	
	Alone	12	0.631–5.917	1.932	0.249
6	Occupation				
	Employed	29	Reference	Reference	
	Unemployed	88	0.544–1.796	0.988	0.970
7	Education				
	School	93	Reference	Reference	
	College and above	16	Reference	Reference	
	No formal education	8	0.304–2.117	0.802	0.657
8	Residence				
	Urban	36	Reference	Reference	
	Rural	81	0.462–1.477	0.826	0.519
9	Status of Hypertension				
	Controlled	40	Reference	Reference	
	Uncontrolled	77	0.708–2.021	1.196	0.503
10	Co morbidities				
	No	56	Reference	Reference	
	Yes	61	0.671–1.884	1.124	0.657
11	Stages of hypertension				
	Normal/Prehypertension	16	Reference	Reference	
	Stage1/Stage 2	101	7.6606–26.801	14.328	0.0001*

*indicates *P* < 0.05 considered as significant.

**Table 4:** Factors associated with depression symptoms in hypertensive patients.

S. No	Parameters	Depression (*n* = 137)	Confidence level	Odds ratio	*p* value
1	Age				
	≤50	23	Reference	Reference	
	>50	114	0.397–1.720	0.826	0.610
2	Gender				
	Male	60	Reference	Reference	
	Female	77	0.823–2.313	1.380	0.222
3	Body mass Index (BMI) (kg/m^2^)				
	<24.9	89	Reference	Reference	
	≥25	48	0.452–1.280	0.760	0.303
4	Marital Status				
	Married	129	Reference	Reference	
	Unmarried	08	0.226–4.338	0.226	0.995
5	Living Arrangement				
	with family	121	Reference	Reference	
	Alone	16	0.612–5.976	1.912	0.265
6	Occupation				
	Employed	33	Reference	Reference	
	Unemployed	104	0.629–2.075	1.142	0.662
7	Education				
	School	106	Reference	Reference	
	College and above	20	Reference	Reference	
	No formal education	11	0.413–2.862	1.087	0.866
8	Residence				
	Urban	34	Reference	Reference	
	Rural	103	0.857–2.705	1.523	0.152
9	Status of Hypertension				
	Controlled	43	Reference	Reference	
	Uncontrolled	94	1.320–3.827	2.248	0.003*
10	Co morbidities				
	No	64	Reference	Reference	
	Yes	73	0.748–2.121	1.260	0.385
11	Stages of hypertension				
	Normal/Prehypertension	28	Reference	Reference	
	Stage1/Stage 2	109	7.4396–24.789	13.58	0.0001*

*indicates *P* < 0.05 considered as significant.

The factors associated with depression among hypertensive patients are presented in Table 4. Uncontrolled BP [OR 2.248, 95% CI (1.320–3.827)] and stages of hypertension (stage1/stage2) [OR 13.58 95% CI (7.4396–24.789)] was found to be significantly associated with depression (*p* < 0.05). However, other factors, such as age >50 years [OR 0.826, 95% CI (0.397–1.720)], female gender [OR 1.380, 95% CI (0.823–2.313)], BMI ≥ 25 [OR 0.760, 95% CI (0.452–1.280)], living alone [OR 1.912, 95% CI (0.612–5.976)], being unmarried or widowed [OR 0.226, 95% CI (0.328–4.338)], unemployed/retired/housewife status [OR 1.142, 95% CI (0.629–2.075)], illiteracy [OR 1.087, 95% CI (0.413–2.862)], living in a rural area [OR 1.523, 95% CI (0.857–2.705)], and having comorbidities [OR 1.260, 95% CI (0.748–2.248)] were not significantly associated with depression (*p* > 0.05).

## 4 Discussion

Anxiety and depression are common in hypertensive patients, and assessing risk factors is essential for early detection and therapy. This study was conducted to evaluate the prevalence and factors associated with anxiety and depression in hypertensive patients at a tertiary care hospital in South India. Among 262 hypertensive patients, the prevalence of anxiety symptoms was 43.8%, while depression was observed in 51.3% of the participants, with over half of the study population experiencing depression.

The prevalence of depression among hypertensive patients in India is reported to be 39.8% (95% CI: 28.6–52.1), with a higher prevalence in South India (44.7%) compared to North India (26.9%) (28). The prevalence of depressive symptoms in the current study was higher compared to studies conducted in other countries, such as Nigeria (29), Ethiopia (30), South Africa (21), and Saudi Arabia (31). Depression is a significant indicator of many long-term medical conditions, including hypertension. It can lead to inadequate self-care, thereby complicating disease management (13).

Over half of the female patients in this study experienced anxiety and depression. Due to biological, life cycle, hormonal, and psychological factors specific to women, females are more likely than males to be diagnosed with depression (20.2% vs. 8.2%). The risk of stroke was highest among women with hypertension and severe depressive symptoms (32). This study also found that individuals over 50 years old were more likely to suffer from anxiety and depression, with depression being nearly twice as common in older adult hypertensive individuals compared to younger ones. Anxiety in the older adult can limit their activities and reduce their quality of life, potentially accelerating the aging process. Therefore, identifying the causes of anxiety is crucial for the early diagnosis and treatment of the condition (33, 34).

The present study showed that anxiety and depression were more common among hypertensive patients who were unemployed, retired, or homemakers. In Indian society, the homemaker or housewife is often the backbone of the household, and the financial and non-financial implications of untreated anxiety extend beyond the individual woman to her family (35). According to the findings of this study, hypertensive patients living in rural areas were significantly more vulnerable to depression than those residing in urban areas. These findings are consistent with a study conducted in China, which found that older adult hypertensive patients in rural areas are at greater risk for depressive symptoms compared to those in urban areas. Although income, exercise, sleep duration, and alcohol consumption were similar in both rural and urban areas, age, gender, education, and alcohol consumption differed among hypertensive patients (36).

Anxiety was significantly higher in patients with uncontrolled BP (65.8%) and depression (68.6%), respectively. The connection between depression and hypertension in hypertensive individuals is not entirely clear, as some medications may worsen depression, and individuals who are depressed may stop taking their prescribed medications, leading to uncontrolled BP (37). People with uncontrolled hypertension frequently experience depression, which may contribute to poor BP control (14). According to a meta-analysis of 41 studies, depression is 26.8% more common among individuals with hypertension. Furthermore, those who suffer from both depression and hypertension are more likely to experience cardiovascular-related morbidity and mortality (38). The current study shows that the stages of hypertension are associated with anxiety and depression. A longitudinal cohort and Mendelian randomization study demonstrated that anxiety symptoms were independent of depression and positively correlated with high blood pressure (39). In contrast to previous studies, the findings of the current study indicated that lower blood pressure among the study population was predicted by symptoms of anxiety and depression (40, 41). According to the current study, there is a strong correlation between anxiety and obesity in individuals with hypertension. Additionally, obesity is associated with an increased risk of depression, metabolic disorders, anxiety, cardiovascular diseases, and chronic inflammatory conditions (42). A meta-analysis found that obese individuals have a 55% higher chance of developing depression over time, while depressed individuals have a 58% higher chance of becoming obese, highlighting the bidirectional relationship between depression and obesity (43). Obesity is strongly linked to two of the most prevalent mental health conditions: anxiety and depression. Conversely, depression and anxiety have also been identified as potential risk factors for obesity (44).

The findings of the current study demonstrated that among patients with hypertension, factors such as female gender, obesity were statistically associated with anxiety and stage 1/stage 2 hypertension as well as depression. Uncontrolled blood pressure was significantly associated with depression. This finding is consistent with studies from Pakistan (45), Mexico (46), and Nigeria (14). According to a meta-analysis, depression is most likely a risk factor for hypertension on its own and should be taken into account when preventing and treating hypertension (37). However, other studies conducted in Ethiopia (47), Pakistan (45), and Saudi Arabia (48) were not consistent with our results. According to the findings of those studies, anxiety and depression in hypertension patients were significantly associated with factors such as age (> 50 years), lower socioeconomic status, lack of formal education, inadequate social support, widowhood or divorce, and comorbid conditions. The disparity in results may be due to variations in study design, methodologies, and the populations studied. Socioeconomic status has been shown to predict depression symptomatology in other study, suggesting that considering SES is an important factor in developing depression prevention strategies (49). While this study did not collect specific data on income or direct measures of social support, related variables such as living arrangements and employment status were included. However, these factors did not show a significant association with anxiety or depression, underscoring the need for more comprehensive data collection in future research. A deeper understanding of how SES and social support influence mental health outcomes could help inform more targeted approaches to managing depression, particularly in populations with chronic conditions such as hypertension.

### 4.1 Limitations

The current study cannot establish causality or the directionality of the relationship between anxiety/depression and hypertension due to the cross-sectional nature. Future research is needed to determine whether untreated mental health issues contribute to the development of hypertension or whether hypertension predisposes individuals to long-term anxiety and depression. Additionally, depression was assessed through a questionnaire, rather than a clinical diagnosis by a psychiatrist. Self-reported symptoms may also be subject to recall bias and influenced by socio-cultural factors. A single BP measurement may not reflect long-term control due to daily fluctuations, and repeated measurements or ambulatory BP monitoring would offer more accurate data. Future studies with continuous monitoring could provide better insights into the relationship between BP control and mental health outcomes. Larger sample sizes or more balanced subgroup distributions could help clarify the potential associations between factors like living alone or low education and mental health outcomes (e.g., anxiety or depression). Further, studies with more specific hypertension severity categorizations could better explore the relationship between hypertension severity and mental health outcomes. The exclusion of patients with pre-existing psychiatric comorbidities may reduce the generalizability of our findings, as many hypertensive patients in real-world clinical settings also experience anxiety or depression. The management and mental health outcomes in such individuals may differ from those without psychiatric conditions. Future research including individuals with known psychiatric disorders would provide a more comprehensive understanding of the mental health burden in hypertensive populations. Future studies should use individual-level designs with comprehensive data collection and advanced analysis to better assess the prevalence of anxiety and depression, and their link to socioeconomic factors, providing a more nuanced understanding of the psychosocial determinants of mental health in hypertensive individuals.

## 5 Recommendations

Based on the findings, certain groups, such as women with hypertension, those with higher BMI, and patients with uncontrolled BP, are at higher risk for anxiety and depression. To prioritize these groups, we recommend closer follow-up with more frequent visits (monthly for uncontrolled BP or high BMI) to monitor both hypertension and mental health, as well as more frequent mental health evaluations through regular screenings for anxiety and depression.

## 6 Conclusion

According to the findings, patients with hypertension frequently developed symptoms of anxiety and depression. Over 50% of hypertension patients reported experiencing these symptoms. The results revealed a significant association between anxiety and depression symptoms with uncontrolled and high blood pressure (stage 1 and 2), female gender and obesity. To implement early screening for anxiety and depression in hypertensive patients, screenings should occur during regular follow-up visits, at least every 6 months for stable patients, and more frequently (every 3–4 months) for those with additional risk factors. Primary care providers, nurses, or clinical psychologists can administer screenings. Integrating this into routine hypertension management can help identify mental health concerns early, improving patient outcomes. Furthermore, special attention is needed for women with hypertension who also have comorbid conditions and lack social support.

## Data Availability

The original contributions presented in the study are included in the article/supplementary material, further inquiries can be directed to the corresponding author.
